# P23 Rates, treatment and outcomes of vancomycin-resistant *Enterococcus faecium* bacteraemia at Addenbrooke’s Hospital

**DOI:** 10.1093/jacamr/dlad066.027

**Published:** 2023-06-14

**Authors:** David Barnes, Beth Blane, Theodore Gouliouris

**Affiliations:** University of Cambridge Clinical School of Medicine, Addenbrooke’s Hospital, Hills Rd, Cambridge CB2 0SP, UK; Department of Medicine, University of Cambridge, Cambridge, UK; Department of Medicine, University of Cambridge, Cambridge, UK; Clinical Microbiology and Public Health Laboratory, Addenbrooke’s Hospital Cambridge, UK

## Abstract

**Background:**

Vancomycin-resistant *Enterococcus faecium* (VRE-fm) bacteraemias represent important nosocomial infections with significant associated mortality and morbidity.

**Objectives:**

To (i) describe the rates, treatments, and outcomes of VRE-fm bacteraemia at Addenbrooke’s Hospital in 2020–21 and compare to a cohort from 2006 to 2012 and (ii) describe rates of antibiotic resistance in VRE-fm bacteraemia isolates from 2015 to 2021.

**Methods:**

71 eligible adult patients with positive VRE-fm blood cultures from 2020 to 2021 were compiled, and electronic patient notes were reviewed retrospectively to obtain microbiological and clinical data. These were compared to a cohort of 187 patients from 2006 to 2012. A nine-person paediatric cohort from 2020 to 2021 was analysed separately. Antibiotic resistance rates were calculated among VRE-fm isolates from blood cultures collected from 2015 to 2021.

**Results:**

A non-significant increase in VRE-fm bacteraemia cases was observed in 2020–2021. Patients were on average, older, with different spectrums of comorbidities. Despite similar Charlson Comorbidity Indices and Pitt Bacteraemia Scores, they also demonstrated a significant reduction in 30-day crude mortality from 34% to 18% (*P* = 0.015). This effect could be accounted for by a lower frequency of risk factors for mortality, such as renal and liver diseases in the 2020–21 cohort. Conversely, treatment with daptomycin was used more commonly among the 2020–21 cohort and was associated with greater survival.

**Conclusions:**

Improved 30-day all-cause mortality in patients with VRE-fm bacteraemia were noted in 2020–21 compared with a historical 2006–12 cohort. High numbers of VRE-fm bacteraemia of nosocomial onset remain a significant concern from both an infection control and antimicrobial stewardship perspective.

